# The Daily Profiles of Circulating AMH and INSL3 in Men are Distinct from the Other Testicular Hormones, Inhibin B and Testosterone

**DOI:** 10.1371/journal.pone.0133637

**Published:** 2015-07-20

**Authors:** Yih Harng Chong, Michael W. Pankhurst, Ian S. McLennan

**Affiliations:** 1 Department of Anatomy, Otago School of Medical Sciences, University of Otago, Dunedin, New Zealand; 2 Department of Medicine, Dunedin School of Medicine, University of Otago, Dunedin, New Zealand; 3 Brain Health Research Centre, University of Otago, Dunedin, New Zealand; University Hospital of Münster, GERMANY

## Abstract

The testes secrete four hormones (anti-Müllerian hormone, insulin-like peptide 3, Inhibin B and testosterone) from two endocrine cell types. It is unknown whether anti-Müllerian hormone and insulin-like peptide 3 levels have a diurnal variation, and if so, whether they covary during the day with testosterone and InhB. Sera were obtained from 13 men at 00:00, 06:00, 09:00, 12:00, 14:00, 17:00 and 19:00 hours and the levels of their testicular hormones measured by ELISA. A second cohort of 20 men was similarly examined with blood drawn at 19:00 and the following 06:00. Anti-Müllerian hormone levels exhibited a subtle diurnal pattern with a 19:00 peak that was 4.9% higher on average than the 06:00 nadir (p = 0.004). The decrease in anti-Müllerian hormone coincided with a rise in testosterone and InhB, but there was no association between the person-to-person variation in the diurnal patterns of anti-Müllerian hormone and testosterone or Inhibin B. Insulin-like peptide 3 had no diurnal pattern, with only minor sporadic variation between time points being observed in some men. In conclusion, the diurnal and sporadic variation of each testicular hormone is distinct, indicating that the major regulation is at the level of the hormone rather than at the endocrine cell type. Consequently, the balance of the hormones being released by the testes has complex variation during the day. The physiological significance of this will vary depending on which combinations of testicular hormones that the target cells respond to.

## Introduction

The testes is a complex endocrine organ, with two endocrine cell types: the Leydig cell that produces testosterone and insulin-like peptide 3 (INSL3) and the Sertoli cell that produces inhibin B (InhB) and anti-Müllerian hormone (AMH). The functions of the three protein hormones (AMH, INSL3 and InhB) are only beginning to emerge, particularly for AMH and INSL3. AMH is a putative regulator of the cardiovascular system [1], with possible relevance to atherosclerosis [2]; INSL3 has emerging roles in spermatogenesis [3] and bone metabolism [4]; whereas InhB is an established regulator of the pituitary [5] and bone [6]. Consequently, the wider testicular influence on men is incompletely understood, despite the wealth of information relating to testosterone.

The mechanisms that underlie acute, chronic and diurnal variations of the testicular hormones appear to be at least partially independent. Consequently, the levels of the testicular hormones may be linked by one form of regulation, whilst varying independently under the influence of another. For example, INSL3 and testosterone levels are both influenced by the number and state of differentiation of Leydig cells, but only testosterone levels vary in response to acute changes in LH [7]. Similarly, AMH and InhB levels in men only partially correlate (R^2^ = 0.24), indicating that the two Sertoli cell hormones are subjected to distinct regulation, whilst also being bounded by lesser common determinants [8].

InhB [9–11] and testosterone [12] have diurnal patterns, which in the case of testosterone are modulated by sporadic determinants such as the onset of REM sleep [13, 14], physical activity [15], timing of meals [16], and social determinants [17]. It is currently unknown whether these mechanisms also affect the levels of AMH and INSL3, as the diurnal pattern of these hormones has not been previously described. We report here that AMH has a subtle diurnal variation whereas INSL3 does not, with both hormones exhibiting minimal sporadic variation.

## Materials and Methods

### Study participants

The study was of community based young men, with no known health issues. Men were recruited from the University of Otago and from the staff of the Dunedin Public Hospital by advertisement. The median age of the men was 26.7 years, with a range of 19.3 to 57.3 years (Table 1). Only one of the men was older than 40 years of age. The men were not taking any regular medications, were non-smokers, had no known testicular or pituitary disorders, were not obese and were not involved in shift work. This project was approved by the University of Otago Human Ethics Committee (Health) and all participants provided informed consent.

**Table 1:** Characteristics of participants in both studies.

	Study 1	Study 2
**Sample size n**	13	20
**Age (years)**	28.7 ± 9.5	25.8 ± 3.2
**AMH (pM)**	47.5 ± 20.4	57.6 ± 23.5
**INSL3 (ng/mL)**	1.6 ± 1.2	2.4 ± 0.9
**T (nM)**	14.9 ± 8.4	20.2 ± 9.2
**InhB (pg/mL)**	156.8 ± 46.9	187.3 ± 68.0
**FSH (IU)**	4.9 ± 1.9	4.7 ± 2.0
**LH (IU)**	3.1 ± 1.9	3.7 ± 1.4
**Cortisol (nM)**	189.5 ± 136.6	

The data are the mean ± standard deviation of the 9 am time of day values for study 1 and the 6 am value for Study 2. 1 ng/ml AMH = 7.14 pM.

### Study 1: blood sampling

Thirteen men had a peripheral intravenous catheter inserted into their antecubital fossa vein at 20:00 for acclimatisation. They were housed either in their own dwelling (n = 6) or slept in a communal university hostel (n = 7). The sleep location had no effect on the hormone levels, and the data for the two groups were combined. Participants were encouraged to maintain their regular routine and sleeping pattern. A series of 6 mL blood samples were aspirated at the following times: 00:00, 06:00, 09:00, 12:00, 14:00, 17:00 and 19:00 by a male doctor. The 06:00 sampling was fasting and pre-dawn, the 12:00 and 17:00 bloods were pre-meal, with the corresponding post-prandial bloods 2 hours later. Sunrise was approximately 06:30 and sunset was approximately 20:50 during the sampling period. The catheter was flushed with isotonic saline periodically to maintain patency. Blood samples were clotted at room temperature for one hour, centrifuged and the serum was aliquoted and snap frozen for storage at -80°C until assay.

### Study 2: blood sampling

A second study was conducted to increase the statistical power and validate observations from study 1. This group consisted of 20 men, 19 new participants and one man from the first study who was re-sampled. Using the methodology above, blood samples were drawn at 19:00 on day 1 and at 06:00 on day 2, with sunset at 19:00 and sunrise at 07:00 during the second study.

### Hormone assays

The levels of hormones were measured using commercial enzyme-linked immunosorbent assay (ELISA), according to the manufacturers’ instructions. The ELISAs were: AMH, (Beckman Coulter, A79765. The assay buffer was added to the serum before addition to the ELISA plate, in accordance with field safety notice FSN-20434-3, June 2013); cortisol (R&D, KGE008); FSH, (ALPCO, 11-FSHHU-E01); InhB, (Beckman Coulter, A81303); INSL3, (Phoenix Pharmaceuticals, FEK-035-27) and LH, (ALPCO, 11-LUTHU-E01). The AMH ELISA measures total AMH (proAMH and AMH_n,c_) [18, 19]. Assays were conducted in duplicate, with quadratic equations or 4-parameter logistic curves (INSL3) used to calculate sample values from the standard curves, according to the manufacturers’ instructions. International reference standards are not available for either INSL3 or AMH, which precludes the comparison of absolute levels of these hormones between studies. Testosterone was measured commercially by the Canterbury Health Laboratory using an in-house ELISA method [20]. In brief, testosterone was extracted from serum using diethyl ether, then detected using a competitive ELISA with a goat anti-rabbit peroxidase labelled antibody against testosterone. The levels of all hormones were above the limit of detection of the ELISAs, with the intra-assay coefficient of variation (CV) being less than 6% for AMH and InhB and less than 10% for INSL3 and testosterone. Each man’s samples for each experiment were assayed at the same time, on the same ELISA plate. The inter-assay CVs were less than 10% for all hormones except for INSL3 that was less than 15%.

### Calculations and statistical analysis

The hormone values for each participant were normalised to their 24-hour mean. This ensures that each participant’s value is of equal weight in the statistical tests, by removing the influence of inter-person variation in the absolute level of the hormones.

The number of possible diurnal patterns is large, which limits direct comparison between time points, unless there is an *a priori* hypothesis about the pattern. Study 1 was therefore tested using repeated measures ANOVA, with pairwise comparison as the posthoc test. Study 1 detects overt diurnal variation, as occurs with cortisol and testosterone, but lacks the power to detect subtle variation. Hence, the second study was designed to test a specific diurnal pattern, with the 06:00 and 19:00 samples being compared using a paired Student t-test.

Covariance between the testicular hormones were tested using mixed model analyses with the hormones as independent continuous variables, and time points as categorical variables [21]. The secretions of gonadotrophins are pulsatile, with the intrapersonal levels varying considerably with a frequency that is shorter than the sampling intervals for the testicular hormones. This precludes any covariant analysis between the pituitary and testicular hormones, in the current study.

The calculations were performed using Stata Statistical Software, Release 13 (StataCorp).

## Results

The 9 am testosterone, InhB, FSH and LH levels for all participants were within the normal range of the local clinical laboratory, with the exception of one individual whose testosterone level was marginally low (8.7 nM, 9–38 nM range). This man was not obese and proven fertility. Normal clinical ranges have not been established for AMH or INSL3 in men. The AMH values of all men were within the reference biochemical range of local men, with defined health status [1]. There is no reference range for INSL3.

**Fig 1:**
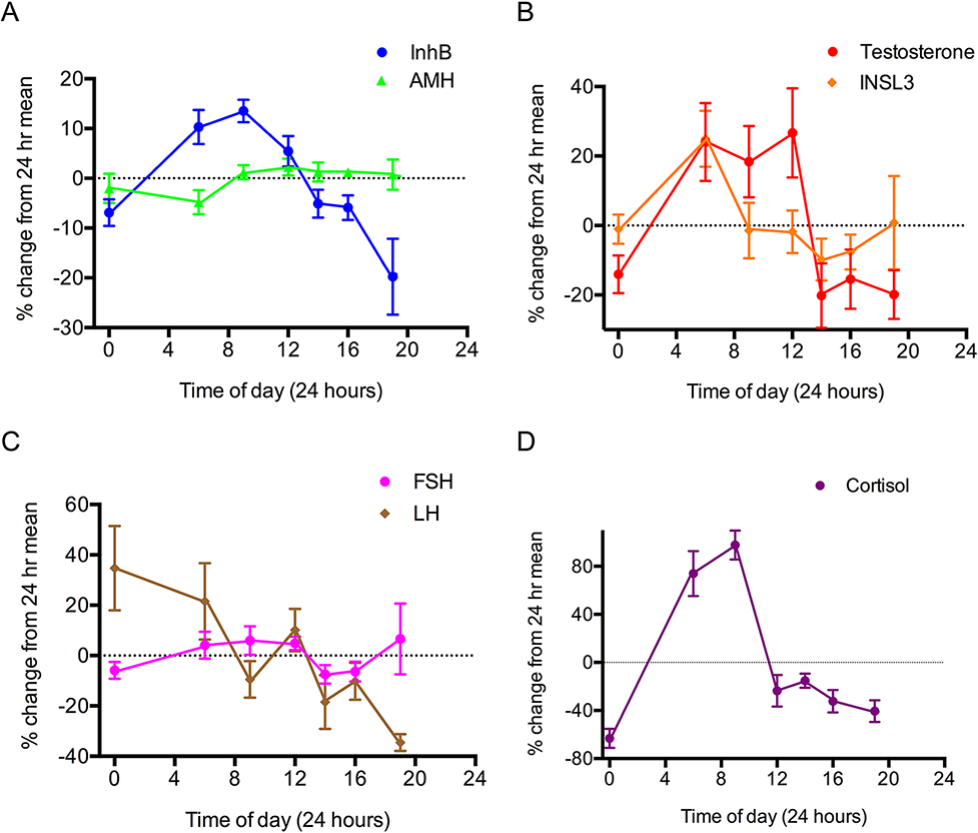
Study 1: diurnal pattern of hormonal expression. Each individual’s hormone levels were normalised to that individual’s mean across the entire sample period. (A) Sertoli cell hormones: InB (blue circles) and AMH (green triangles); (B) Leydig cell hormones: testosterone (red circles) and INSL3 (orange diamonds); (C) Gonadotropins: LH (brown diamonds) and FSH (pink circle) and (D) Circadian marker: Cortisol. The single 0:00 data point is plotted at both 0:00 and 24:00 with the dashed line used to indicate this extrapolation. The data are the mean ± the standard error of 13 men.

All men in Study 1 exhibited a normal daily pattern of cortisol (Fig 1D) [22], verifying that their endogenous circadian rhythm was intact. LH had a significant diurnal variation as expected [23, 24], with higher values in the nocturnal and early morning periods, and subsequently trending towards an evening nadir (F(6,12) = 3.95, p = 0.032) (Fig 1C). FSH, in contrast, did not vary significantly between time points (F(6,12) = 1.05, p = 0.36) (Fig 1C). Similar results were obtained for LH and FSH in a validation experiment (Study 2) comparing 6:00 and 19:00 values in 20 men (LH 6:00 4.7 ± 0.5 IU vs 19:00 3.7 ± 0.3 IU, p = 0.032 paired student t test; FSH 6am 4.1 ± 0.5 IU vs 7 pm 4.0 ± 0.4 IU, not significantly different).

### Sertoli cell hormones

**Fig 2:**
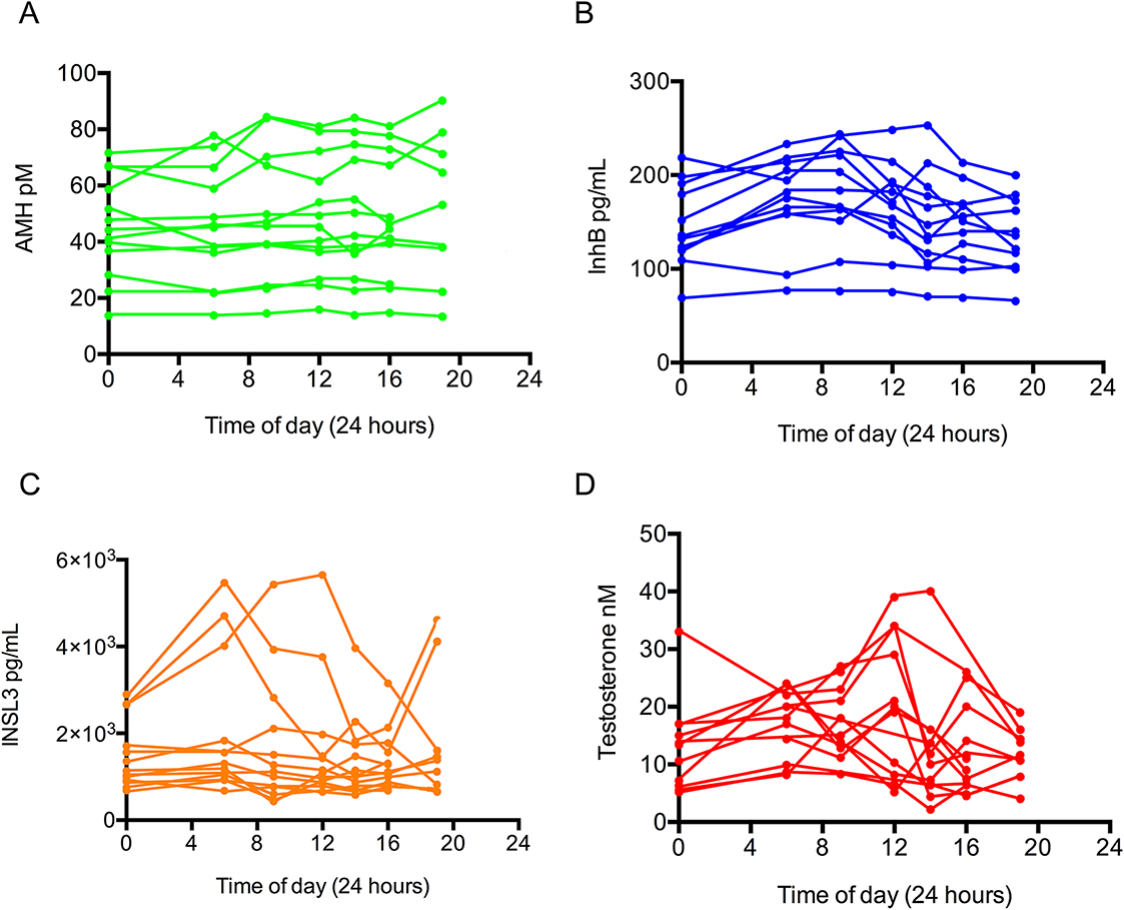
Study 1: Daily trend of serum testicular hormones from 13 healthy men plotted individually. (A) AMH, (B) InhB, (C) INSL3 and (D) testosterone. The 00:00 data points are plotted at both 0:00 and 24:00, with the dashed line used to indicate this extrapolation.

**Fig 3:**
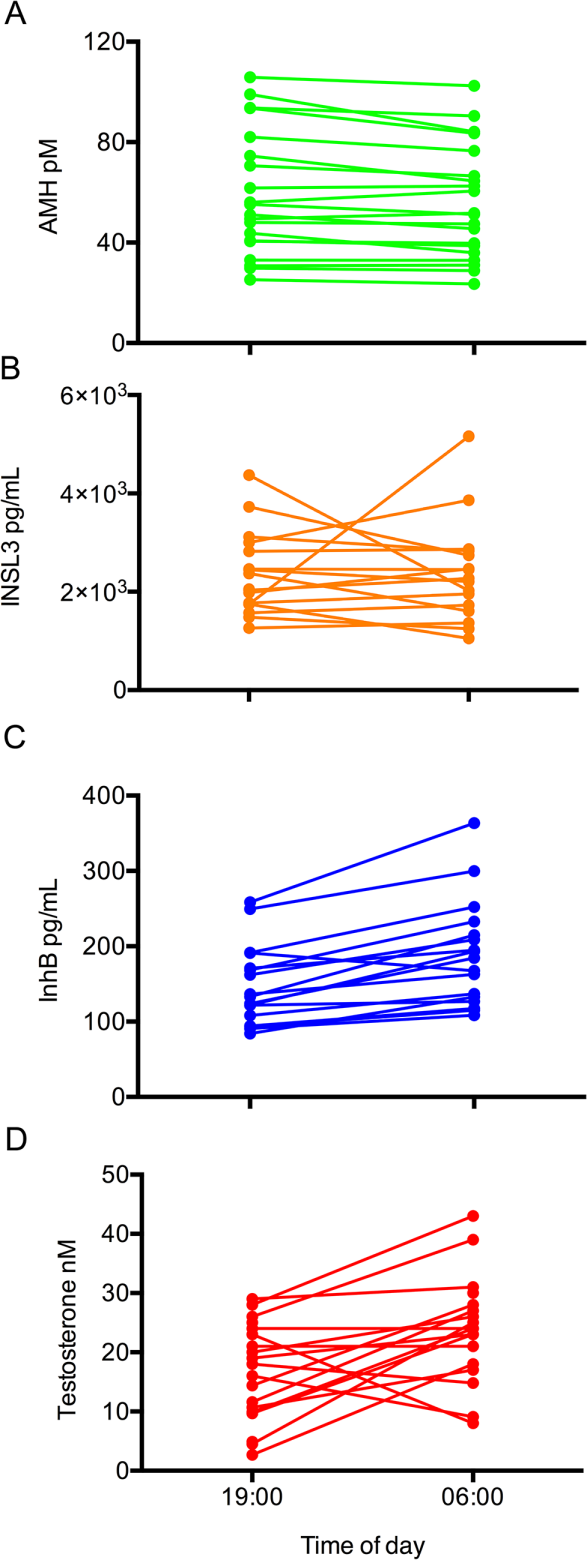
Changes in serum testicular hormonal concentration between 19:00 and 06:00 (Study 2). (A) AMH, (B) INSL and (C) InhB and (D) testosterone levels were analysed in 20 men. AMH, on average declined by 4.9 ± 1.8%, (p = 0.004, paired Student t-test), whereas the levels of InhB and testosterone increased on average by 28 ± 4% (p<0.0005) and 41% (p = 0.011) respectively. There was no significant change in the levels of INSL3, on average (p = 0.78). The mean levels of the hormones at 19:00 and 06:00 were: AMH (57.9 ± 5.7, 55.0 ± 5.3 pM); InhB (147 ± 13, 189 ± 17 pg/ml); INSL3 (2.28 ± 0.20, 2.21 ± 0.24 ng/ml); testosterone (16.0 ± 1.9, 24.5 ± 2.0 nM).

There was no statistical variation in AMH across the day in study 1 (F(6,12) = 0.69, p = 0.48), indicating the absence of a consistent and overt diurnal profile. However, AMH levels were moderately lower in the nocturnal phase, with a nadir at 06:00, and a subsequent rise in the hours after waking (Figs 1A and 2A). The veracity of this subtle variation was tested in a larger cohort of 20 men, with a target comparison between their 06:00 and 19:00 values. This confirmed that the 06:00 levels were slightly lower on average (4.9%, p = 0.004, paired Student t-test), with the inter-person variation encompassing a range between -7.9% and 18.1% (Fig 3).

In contrast, the other Sertoli cell hormone InhB exhibited an overt diurnal pattern with significant variation throughout the day (Fig 1A, F(6,11) = 10.67, p = 0.000). The InhB values peaked at 09:00 and then diminished to a 19:00 nadir, with an average total decrease of 26% during this period. This diurnal pattern was observed in 11 of the 13 participants, with the levels of InhB in the other two men being relatively stable throughout the day (Fig 2B).

### Leydig cell hormones

INSL3 was the least variable testicular hormone with no significant variation observed across the study population (Fig 1B, F(6,12) = 2.10, p = 0.13). The moderate spike in INSL3 observed at 06:00 was predominantly caused by variable levels in 3 individuals with no consistent circadian pattern (Fig 2C). When each man’s values were normalised to his average value for the day, the standard deviation for all the data points was 26%. There was also no significant difference on average in INSL3 levels in Study 2 (p = 0.78 paired T-test), although 4 of the 20 men had non concordant values at 06:00 and 19:00, but without consistent direction; two had higher values at 06:00 and two had lower (Fig 3B).

Serum testosterone levels in Study 1 were consistent with the established diurnal profile [12], with a peak morning value and a nadir concentration in the evening (Fig 1B, F(6,12) = 4.52, p = 0.040). There was an average decrease of 47% between the 06:00 and the19:00 nadir samples. This circadian pattern was replicated in Study 2 with an average 41% increase between 19:00 and 06:00 the following day (Fig 3C, p = 0.011, paired Student t-test). The average amplitude for the testosterone diurnal variation was larger than that of InhB (47% vs 26%, respectively), but with less statistical confidence because the testosterone rhythms were highly variable between individuals.

### Hormone covariations

The time-point to time-point variation in InhB partially correlated to testosterone only (mixed model covariation **β** coefficient = 1.26, p = 0.001) but not to AMH or INSL3 (Table 2). AMH, INSL3 and testosterone did not covary with each other. The absence of correlation was also evident when every individual was examined. For example, the individuals with the most overt changes in AMH levels did not have the largest changes in testosterone. Similarly, the individuals with some variation in their levels of INSL3 had unremarkable diurnal variations in testosterone, InhB, relative to other men.

**Table 2:** Covariation of the diurnal patterns of the hormones.

	InhB	AMH	Testosterone
**InhB**	-	0.06 (p = 0.48)	1.26 (p = 0.001)*
**AMH**	0.06 (p = 0.48)	-	-0.30 (p = 0.65)
**Testosterone**	1.26 (p = 0.001)*	-0.30 (p = 0.65)	-
**INSL3**	0.09 (p = 0.049)*	0.03 (p = 0.43)	0.07 (p = 0.68)

The covariation between time points for each of the testicular hormones were calculated for 20 men using mixed model analysis, and expressed as the covariation **β** coefficient (p value). Note that the data is normalised to the daily mean, and is therefore independent of the absolute levels of the men’s hormones.

* indicates statistical significance.

## Discussion

AMH has a subtle diurnal variation with an evening peak whereas INSL3 exhibits no overt diurnal pattern. Hence, the Leydig and Sertoli cells each secrete one hormone with minimal variation during the day (INSL3 and AMH) and another hormone with an overt diurnal pattern (testosterone and InhB), indicating that the diurnal variation operates at the level of the hormone and is not a universal property of the endocrine cell type.

The circulating level of a hormone is a product of its synthesis, release and clearance. Testosterone and InhB, which had the most covariance, have similarly short half-lives in blood [25, 26]. AMH, in contrast, has a long half-life that is not fully characterised in men. In women and bulls the half-life of AMH exceeds one day [27, 28], with the mechanism by which AMH is cleared from blood being unknown. Consequently, the presence of a subtle change in the levels of AMH between 06:00 and 19:00 (Fig 3) may represent a larger diurnal variation in either the production or the secretion from the testes, with the magnitude of the change in serum levels being dampened by its slow clearance.

The synthesis of AMH is putatively decreased by testosterone [29], although anti-androgen therapy does not change plasma AMH levels [30]. The diurnal decrease in AMH coincided with a diurnal rise in testosterone, raising the possibility that the variation in AMH is secondary to the larger diurnal variation in testosterone. However, both AMH and testosterone cycles vary in magnitude and direction between men. Consequently, if the daily pattern of AMH levels is driven by testosterone then the person-to-person variation in the two hormones should associate, which was not the case. This suggests that testosterone is not the main driver of the daily variation in AMH levels.

INSL3 is an emerging biomarker for hypogonadism, with the greater day to day consistency of INSL3 than testosterone being advantageous in this context [3, 4]. The absence of diurnal variation in INSL3 further supports the use of INSL3 as a biomarker for Leydig cell function. The overall intraperson variation in INSL3 levels between points was 26%, which is similar to the 21% day-to-day variation reported by Ivell et al [3]. These figures include variation from the ELISA, indicating that the level of sporadic variation in INSL3 levels is less than 10%. This is in marked contrast to testosterone which exhibits sporadic variation, due to multiple environmental determinants (see Introduction). This not withstanding, we emphasise that a minority of men exhibit transient elevations of INSL3, which are consistent with episodic variation in either the release or clearance of INSL3, with the frequency between episodes being greater than 1 day. The levels of INSL3 observed in cross-sectional studies typically exhibit a right-skewed distribution [31](see also younger men in [32]). The significant prevalence of men with higher levels of INSL3 in these studies may in part be explained by an episodic release of INSL3 preceding blood sampling.

The diurnal variation in InhB was more pronounced than for AMH, making it a less reliable biomarker of Sertoli cells. There was partial covariance in the diurnal rhythm of InhB and Leydig-derived testosterone, which has been reported in some but not all previous studies [33, 34]. This is consistent with diurnal variation in the testes being driven by a common circadian influence on both its endocrine cell types, with the pattern not being universally apparent in all men and on all days due to sporadic influences.

All men in the study had normal levels of FSH, LH and cortisol, indicating the presence of a normal circadian rhythm and the absence of overt pituitary dysfunction. LH and FSH levels are pulsatile [23], and high frequency blood sampling is required to study their functions. The current study is therefore uninformative of whether gonadotrophins contribute to the sporadic or diurnal variation in the testicular hormones. One individual had a testosterone value that was marginally outside of the local clinical range. The diurnal variations of this individual’s hormones did not differ from the other participants. He was included in the study, as the design was community based.

## Conclusions

In conclusion, each of the four testicular hormones has a distinct diurnal pattern and sporadic variation, despite their common cellular origins and the influence of shared regulators. Consequently, the relative levels of the four hormones within an individual has a complex daily profile, with the physiological significance of this being currently unclear, due to the paucity of information about the non-reproductive actions of the testicular protein hormones.
